# Detection by metagenomic functional analysis and improvement by experimental evolution of β-lactams resistance genes present in oil contaminated soils

**DOI:** 10.1038/s41598-022-13883-x

**Published:** 2022-06-29

**Authors:** M. Teresa Álvarez-Marín, Laura Zarzuela, Eva M. Camacho, Eduardo Santero, Amando Flores

**Affiliations:** Departamento de Biología Molecular e Ingeniería Bioquímica, Centro Andaluz de Biología del Desarrollo, CSIC, Universidad Pablo de Olavide, Junta de Andalucía, Carretera de Utrera, Km. 1, 41013 Sevilla, Spain

**Keywords:** Microbiology, Antimicrobials, Environmental microbiology

## Abstract

The spread of antibiotic resistance genes has become a global health concern identified by the World Health Organization as one of the greatest threats to health. Many of antimicrobial resistance determinants found in bacterial pathogens originate from environmental bacteria, so identifying the genes that confer resistance to antibiotics in different habitats is mandatory to better understand resistance mechanisms. Soil is one of the most diverse environments considered reservoir of antimicrobial resistance genes. The aim of this work is to study the presence of genes that provide resistance to antibiotics used in clinical settings in two oil contaminated soils by metagenomic functional analysis. Using fosmid vectors that efficiently transcribe metagenomic DNA, we have selected 12 fosmids coding for two class A β-lactamases, two subclass B1 and two subclass B3 metallo-β-lactamases, one class D β-lactamase and three efflux pumps that confer resistance to cefexime, ceftriaxone, meropenem and/or imipenem. In some of them, detection of the resistance required heterologous expression from the fosmid promoter. Although initially, these environmental genes only provide resistance to low concentrations of antibiotics, we have obtained, by experimental evolution, fosmid derivatives containing β-lactamase ORFs with a single base substitution, which substantially increase their β-lactamase activity and resistance level. None of the mutations affect β-lactamase coding sequences and are all located upstream of them. These results demonstrate the presence of enzymes that confer resistance to relevant β-lactams in these soils and their capacity to rapidly adapt to provide higher resistance levels.

## Introduction

Very soon after their discovery, antibiotics became one of the major achievements in the history of medicine. However, the widespread use and misuse of antibiotics in human and veterinary medicine and agriculture in the last decades have led to the acquisition and spread of antibiotic resistance genes (ARG) among pathogens, which has reduced the effectiveness of these compounds. Nowadays, this issue represents a relevant public health threat, leading to societal and economic impacts, which has prompted the development of global action plans to combat antibiotic resistance^1–3^. In this regard, elucidating the origin of these ARGs is an essential objective that will allow predicting and fighting the appearance of new resistant bacteria.

Among antimicrobial resistance (AMR) determinants, β-lactamases are the most spread cause of bacterial resistance to β-lactam antibiotics. These enzymes are classified into four different molecular classes according to the nucleotide and protein sequences, from A to D^4^. Additionally, β-lactamases have been divided into two biochemical groups based on the hydrolysis mechanism: classes A, C and D are serine β-lactamases^5^ while class B are metallo-β-lactamases (MBLs) which require one (class B2) or two zinc ions (classes B1, B3 and B4) to facilitate β-lactam hydrolysis^6,7^.

On account of their predominance in clinical isolates, most research has mainly focused on class B MBL^8,9^. This concern is also based on their insensitivity to β-lactamase inhibitors and its capacity to confer resistance to carbapenems, last-resort β-lactam antibiotics used in severe bacterial infections treatments^10^. On the other hand, class D are the most diverse enzymes among the four β-lactamase classes at both biochemical and genetic level, and in relation to their antibiotic spectrum, thus comprising enzymes with a narrow or expanded spectrum and providing resistance to different antibiotics from penicillins to carbapenems^11^.

Many of the AMR genes carried by bacterial pathogens of clinical relevance have originated from environmental bacteria^12–15^. Sometimes, these environmental genes exert an antibiotic protective function but others have a different role. Soil, one of the wider and varied microbial habitats with the highest diversity of prokaryotes, is considered an important reservoir and source of antibiotic resistant bacteria and AMR determinants. These genes can potentially spread through different ecosystems and be transferred to human and animal pathogens by horizontal gene transfer, which is a major concern. Some of these genes are likely origin of ARGs of high clinical relevance^13,16–18^, but their detection and study is hampered since fewer than 1% of environmental species of microorganisms are currently cultivable^19^. However, sequence-based studies and functional metagenomic approaches have enabled an increasing amount of research focused on AMR determinants in soils and other environments, which have revealed the presence, in all these habitats, of many known and novel resistance genes^20–25^. These studies have mostly focused on soils as diverse as pristine sites or soils affected by different types of human activities but little is known about the presence of resistance genes in oil contaminated soils. Additionally, it has been reported the correlation of the abundance of some AMR determinants with the presence of metals in oil contaminated soils due to co-selection pressures^26,27^, but the occurrence of AMR determinants has not been properly studied. This co-selection is caused by cross-resistance or co-resistance mechanisms (reviewed in^28^). Cross-resistance can occur when a single mechanism provides resistance to different compounds simultaneously, such as efflux pumps. On the other hand, co-resistance results when different resistance genes are placed on the same genetic element, such as a plasmid, transposon or bacterial chromosome. Therefore, studying the presence of AMR determinants in oil contaminated soils could provide additional information on the distribution of these genes in different environments.

In previous work in our laboratory, two metagenomic libraries were constructed with bacterial DNA from two crude oil contaminated soils in southern Spain, one from an oil refinery and another from a beach, which have been used to detect contaminant-degrading enzymes^29,30^. To overcome the frequent inability of the *E. co*li host strain to express metagenomic genes, the libraries are based on a fosmid vector with a salicylate (Sal) inducible promoter that drives the expression of the metagenomic DNA, even through transcription termination signals, using also the N anti-termination protein from lambda phage. Furthermore, in order to raise gene dosage and consequently, protein production levels, the fosmid copy number can be increased by the addition of arabinose (Ara) to culture medium. In this paper, we report on the selective screening of both libraries for AMR determinants that provide resistance to diverse antibiotics used in clinical settings, some of them as last resorts against infections. Thus, we have isolated fosmids encoding efflux pumps, two class A β-lactamases, one class D β-lactamase, two subclass B1 and two subclass B3 MBLs conferring resistance to cephalosporins and/or carbapenems. Although normally environmental genes confer resistance to low concentrations of antibiotics^31^, experimental evolution assays of the *E.coli* host strain bearing fosmids that encode class B MBLs led to the selection of mutant fosmids with increased resistance level. All of them carried mutations that augmented the β-lactamase activity without changes in their ORFs. The results reveal the presence of relevant enzymes in oil contaminated soils that, once transferred to human related bacteria, could rapidly evolve and provide resistance to high concentrations of clinically important antibiotics and be the origin of the resistance of pathogen species. This shows the importance of studying the occurrence and distribution of these determinants in different environments.

## Results

### Detection of antibiotic resistance genes in two environmental metagenomic libraries

As stated above, we have previously constructed two metagenomic libraries from soils contaminated with crude oil in a fosmid vector that promotes heterologous expression of metagenomic DNA after Sal induction in the specialized strain MPO554. Both libraries have been used to search for contaminant-degrading enzymes and, additionally, to screen for genes conferring carbenicillin (Cb) resistance^30,32^. The presence of fosmids encoding β-lactamases and efflux pumps that provide this resistance prompted us to screen them for fosmids bearing resistance genes to antibiotics mainly used in clinical settings.

Environmental concentrations of antibiotics are usually under detection limits and not in the minimum inhibitory concentration (MIC) range for most environmental bacteria^31^. Therefore, in order to detect also fosmids that provide resistance to low concentrations of antibiotics, we first determined the lowest concentration of each antimicrobial that prevent colony growth of the specialised host strain MPO554Nal^R^ on LB-agar solid media. These concentrations were subsequently used in screens to select for fosmids that confer resistance to antibiotics. Antibiotics studied and concentrations used in screens were the fluoroquinolones norfloxacin 0.08 μg/ml (Nor), ofloxacin 0.5 μg/ml (Ofl) and levofloxacin 0.3 μg/ml (Lev); the polymyxin colistin 5 μg/ml (Col); the cephalosporins ceftriaxone 0.15 μg/ml (Ctr) and cefixime 1.5 μg/ml (Cem) and the carbapenem meropenem 0.1 μg/ml (Mer).

To accomplish screens, we transferred the libraries to the specialized MPO554Nal^R^ strain used for the screening (see Material and Methods), and the transconjugants were selected on LB agar plates supplemented with Ara, Sal and the defined concentrations of the clinical antibiotics. The conjugal transfer frequency of the fosmid clones was 5–10% of the recipient cells and the total of transconjugants were over 1.5 × 10^6^ in each mating. The isolation and analysis of the clones from each antibiotic screening revealed clones resistant to Ctr or Cem in the library from the beach, and to Ctr, Cem or Mer in the library from the oil refinery. Conversely, we did not obtain any fosmid conferring resistance to Nor, Ofl, Lev or Col. After restriction analysis of the fosmids, we chose one representative of each restriction pattern for further studies. We selected four fosmids (CTR4, CTR6, CTR8 and CTR15) resistant to Ctr, and three fosmids (CEM3, CEM4 and CEM6), which confer resistance to Cem from the metagenomic library from beach. Also, we isolated one Ctr resistant fosmid (CTRC-R2), three Cem resistant fosmids (CEMC6, CEMC18 and CEMC19), and one Mer resistant fosmid (MERC5) from the library from the refinery (Table 1).

**Table 1:** Independent clones isolated in the screenings.

Source	Clone	Isolation resistance	Cross resistance	AMR mechanism
Refinery	CTR4	Ctr	Nd	blaSHV
	CTR6	Ctr	Cem	Efflux pump
	CTR8	Ctr	Cem	
	CTR15	Ctr	Nd	blaTEM
	CEM3	Cem	Nd	blaSHV, blaTEM
	CEM4	Cem	–	B3 MBL
	CEM6	Cem	–	
Shore	CTRC-R2	Ctr	Cem	Efflux pump
	CEMC6	Cem	–	B1 MBL
	CEMC18	Cem	Ctr	D β-lactamase
	CEMC19	Cem	Ctr, Mer	B1 MBL
	MERC5	Mer	–	Efflux pump
	TN2	Cb	Ctr, Cem, Mer, Imi	B3 MBL

Antibiotic resistance conferred and resistance mechanism encoded by fosmids.

*Nd* not determined, – no cross resistance.

### AMR determinants selected are β-lactamases and efflux pumps

Since all of the clones detected resist to β-lactam antibiotics, we PCR analysed the presence of different groups of known β-lactamase genes in these fosmids with a collection of standard primers reported in the literature (Table S2, supplemental material). The PCR-analysis revealed that two fosmids, CTR4 and CTR15, carry the blaSHV and blaTEM β-lactamases (class A β-lactamases) encoding genes respectively, and fosmid CEM3 contains both genes. These three fosmids were not used for further analysis. None of the remaining fosmids yielded DNA amplification by PCR with any of the primers, suggesting that they must carry different β-lactamase genes or distinct AMR mechanisms. These fosmids were sequenced and subsequent analysis showed that they all bear either β-lactamase or efflux pumps coding sequences.

**Figure 1:**
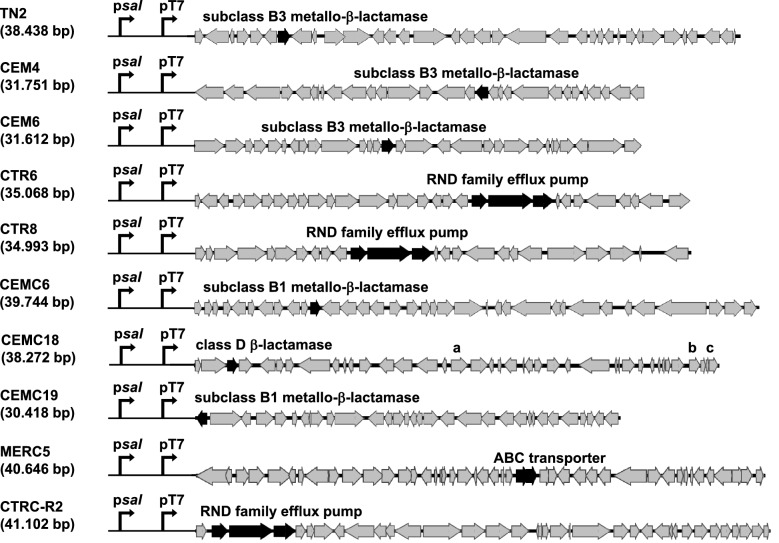
Scheme of the clones that confer resistance to β-lactams antibiotics. All putative *orfs* and their orientation are shown by arrows, which are black for those genes likely involved in the resistance. (**a**) IS4 family transposase. (**b**,**c**) Site-specific integrases.

The structure of the metagenomic sequences and the putative ORFs responsible for the resistance are shown in Fig. 1 and Table 1. Fosmids CEM4 and CEM6 share most of their insert DNA and bear the same subclass B3 MBL. Interestingly, these two clones were identical to N5 and N6 formerly identified in our laboratory in a screening of this library searching for Cb resistant formids^29^. CTR6 and CTR8, share part of their metagenomic DNA sequence and encode the three components of an efflux pump of the resistance-nodulation-cell division (RND) permease family. Likewise, both fosmids were identical to TN3 and TN4 previously isolated as resistant to Cb^29^. CEMC18 codes for a class D β-lactamase while CEMC6 and CEMC19, carry two different subclass B1 MBLs. On the other hand, CTRC-R2 and MERC5 fosmids encode different efflux pumps, but no β-lactamases coding sequences are present in the metagenomic DNA of these latter fosmids.

In a previous work, Terrón et al.^29^ subcloned the efflux pump ORFs present in CTR6 and CTR8 fosmids into the original empty vector to demonstrate that these actually were the sequences responsible of the AMR. We have used the same constructs to confirm that these efflux pumps also provide resistance to Ctr. With the same purpose, we subcloned the efflux pumps ORFs from CTRC-R2 and MERC5 and, additionally, the β-lactamases coding sequences found in this work; all subclones were able to confer resistance to their corresponding antibiotics.

Another fosmid, TN2, was also isolated by Terrón et al. from the shore metagenomic library as resistant to Cb^29^. Although it was not detected in the screens performed in this work, we blast analysed its sequence and the result indicated that it contains a subclass B3 MBL. Additionally, we experimentally observed that it also provides resistance to Cem and Ctr and, therefore, we decided to include TN2 for further studies together with the other β-lactamase coding fosmids detected in the new screens.

Although the β-lactamases identified show similarity with other previously detected in sequencing projects or metagenomic studies, ranging from 70 to 99% of amino acid identity (Table 2), their functionality had not been tested or their resistance to the antibiotics used in this work had not been analysed (Table 2). Therefore, obtaining experimental data on their function is of particular interest.

**Table 2:** Host of best hit homologs of five β-lactamases detected in oil contaminated soil microbiota by functional metagenomics.

Fosmid	AMR gene	Host homologs	Amino acid identity (%)	Accession number
CEM4	subclass B3 MBL	*Acidobacteria bacterium*	70.49	PYR04691
CEMC6	subclass B1 MBL	*Chryseobacterium cucumeris*	99.17	WP_123279817.1
CEMC18	class D β-lactamase	*Legionella massiliensis*	90.62	WP_043873091.1
CEMC19	subclass B1 MBL	*Chryseobacterium sp. CBTAP 102*	93.62	WP_110366687.1
TN2	subclass B3 MBL	uncultured bacterium	86.87	AGD93225.1

### β-lactamases phylogeny and analysis of their AMR determinant genetic context

**Figure 2:**
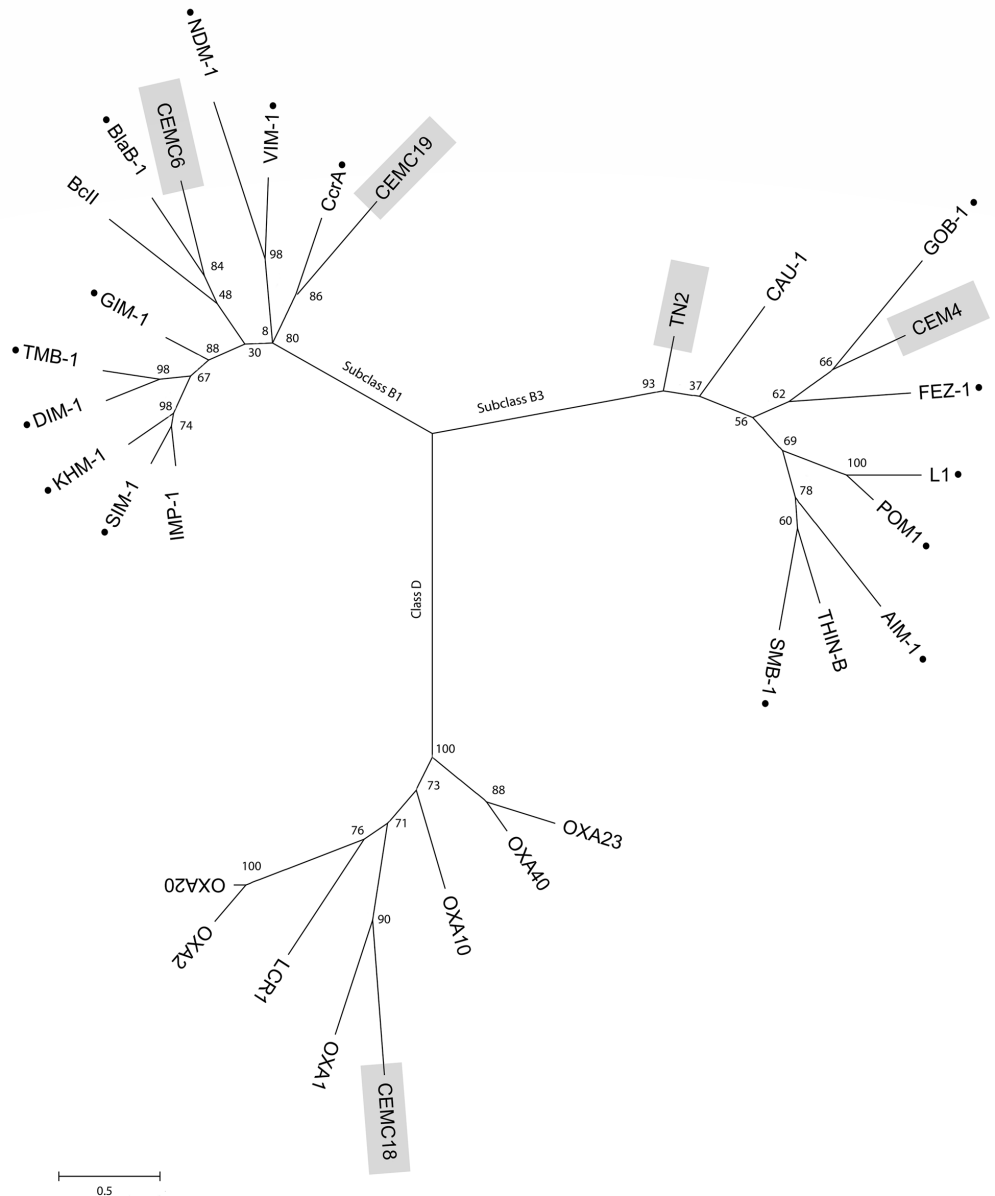
Un-rooted phylogenetic tree of subclass B1 and B3 MBLs and class D β-lactamases. The evolutionary distances are expressed in amino acid substitutions per position. The new MBLs detected in this study are boxed. The MBLs identified in clinical bacteria are indicated by a black dot. Numbers at clades represent their bootstrap confidence levels. Genebank accession numbers of the previously sequenced β-lactamases included in the phylogenetic tree are shown in Supplemental Information.

Additionally, we studied the phylogeny of the five β-lactamases identified in the screens, TN2, CEM4, CEMC6, CEMC18 and CEMC19 and representative members of class D and subclass B1 and B3 β-lactamases. CEM6 was not used in these analyses because it contains the same β-lactamase as CEM4. Phylogenetic tree analysis confirmed that CEMC6 and CEMC19 are MBLs belonging to subgroup B1, TN2 and CEM4 belong to subgroup B3 MBL, and CEMC18 corresponds to class D β-lactamase group (Fig. 2). Specifically, they branch in the same group as BlaB-1 (43.98% of identity), CcrA (39.98% of identity), CAU-1 (46.67% of identity), GOB-1 (43.25% of identity) and OXA-1 (41.53% of identity), respectively. Furthermore, amino acid alignment showed that all zinc-binding amino acids present in subclass B1 and B3 enzymes are conserved (Supplementary Fig. S1).

To further explore the precedence of the resistances, we analysed the genetic context of genes to look for mobile elements that could lead to DNA rearrangements or the spread of resistance through horizontal gene transfer. We detected ORFs showing homology with these sequences only in CEMC18 fosmid, namely, genes for one insertion sequence transposase belonging to IS4 family, one site specific integrase and one tyrosine-type recombinase/integrase (Fig. 1a–c genes, respectively).

On the other hand, comparison of the GC content of AMR determinants and their respective genetic backgrounds showed no differences, thus providing no clues about a possible acquisition from different microorganisms (data not shown).

### MIC analyses show that heterologous expression contributes to increase resistance level

To validate the effective antibiotic resistance of the selected clones, fosmids were analysed by MIC assays of Cem, Ctr, Mer and Imi against specialised MPO554Nal^R^ strain harbouring the fosmids or an empty vector (Table 3). All the analyses were performed either in the presence of Ara plus Sal or in their absence. As expected, all fosmids increased the MIC of the antibiotics for which they were selected. Interestingly, most of the fosmids also increased the MIC for some of the other antibiotics, with the sole exception of MERC5, which showed only resistance to Mer. On the other hand, TN2 was the only fosmid conferring resistance to all antibiotics tested. CEMC18 did not provide resistance to carbapenems, which was expected since its β-lactamase is close to OXA-1 subgroup of class D β-lactamases, which do not confer resistance to carbapenems. However, the B3 MBL encoded in CEM4 or CEM6 was also unable to provide resistance to carbapenems.

**Table 3:** Minimum inhibitory concentration (MIC) of MPO554 strain expressing AMR determinants.

Fosmid	MIC (μg/ml)							
	CEM		CTR		MER		IMI	
	+ Sal + Ara	-Sal-Ara	+ Sal + Ara	-Sal-Ara	+ Sal + Ara	-Sal-Ara	+ Sal + Ara	-Sal-Ara
Empty Vector	0.5	0. 5	0.06	0.06	< 0.125	< 0.125	< 0.125	< 0.125
TN2	**4**	**0.5**	**2**	**0.25**	**0.5**	< 0.125	**0.125**	< 0.125
CEM4	**4**	**0.5**	**0.25**	**0.25**	< 0.125	< 0.125	< 0.125	< 0.125
CEMC6	**8**	**8**	0.06	0.06	< 0.125	< 0.125	< 0.125	< 0.125
CEMC18	**8**	**4**	**1**	**0.25**	< 0.125	< 0.125	< 0.125	< 0.125
CEMC19	**8**	**8**	**2**	**2**	**0.25**	< 0.125	< 0.125	< 0.125
CTR6	**4**	**0.5**	**2**	**0.125**	< 0.125	< 0.125	< 0.125	< 0.125
CTRC-R2	**4**	**2**	**2**	**1**	< 0.125	< 0.125	< 0.125	< 0.125
MERC5	0.5	0.5	0.03	0.06	**0.125**	< 0.125	< 0.125	< 0.125

Bold values correspond to fosmids and conditions showing resistance to antibiotics.

Regarding the MIC of Cem and Ctr, the resistance levels of CEMC6 and CEMC19 fosmids were independent of the addition of Ara and Sal to the medium, indicating that transcription from their own promoters is sufficient to achieve their corresponding MICs. The remaining fosmids provided higher resistance level than the empty vector in the absence of Ara and Sal, but this level increased in their presence.

In the case of the carbapenem resistance, only TN2, CEMC19 and MERC5 provided resistance to Mer and only TN2 provided it to both Mer and Imi. In all these cases, the addition of Ara and Sal was necessary to show the resistance phenotypes, since in their absence, the strains bearing these fosmids were as sensitive as the control carrying the empty vector.

### Experimental evolution leads to the selection of fosmids with increased resistance levels

It is likely that the soils used to construct the metagenomic libraries are poorly exposed to the antibiotics used in this work. In addition, although endogenous and/or heterologous promoters can drive the environmental DNA transcription, transcription and translation by the host *E. coli* strain may not be efficient enough. This could explain why the fosmids selected in the screens confer low levels of resistance, particularly to carbapenems. To test whether prolonged antibiotic exposure could increase the level of resistance, we grew MPO554Nal^R^ carrying different fosmids in serial cultures in the presence of increasing concentrations of antibiotic. Increased resistance to Cem or Ctr were tested with the fosmids CEM6, CEMC18, CEMC19 and TN2, which carry β-lactamase ORFs. The resistance to Mer was assayed with MERC5, which encodes an efflux pump. Cultures of these strains in the presence of the corresponding antibiotic plus Sal and/or Ara were diluted 1/100 in the same medium with increasing concentrations of antibiotics. As a control, we used the same strain carrying the empty vector, pMPO579. Assays were stopped at different final antibiotic concentrations and number of generations. Initial antibiotic concentrations were those used in the screens and final concentrations achieved were between 35 and 55 μg mL^−1^ for Cem, and between 20 and 50 μg mL^−1^ for Ctr. Depending on the fosmids, the estimated number of generations ranged from 63 to 182. After that, bacteria were plated on LB supplemented with the appropriate antibiotic to isolate resistant clones, and to test whether the resistance increase was due to mutations in the fosmids, we transferred the fosmids to the isogenic MPO554Gm^R^ strain by mating. One subculture of CEMC18 grown in presence of Ctr, one of TN2 in presence of Cem and one of MERC5 in presence of Mer reached higher concentrations of the corresponding antibiotic. However, none of them conferred higher antibiotic resistance when they were transferred by conjugation, which could indicate that the resistance increase could be due to mutations in the chromosome of these strains. On the other hand, we obtained two mutant fosmids which provided increased resistance to Cem (called CEMC18-Cem and CEMC19-Cem) and two mutant fosmids conferring resistance to higher concentrations of Ctr (called CEMC19-Ctr and TN2-Ctr).

These four fosmids increased the MIC of all clinical β-lactams used in this work from 2 to 64-fold (Table 4), with the exception of CemC18-Cem, whose β-lactamase is not a carbapenemase. CEMC18-Cem and TN2-Ctr increased their resistance level in the presence of Sal and Ara. This result is in agreement with the fact that expression of these β-lactamases are driven by both its own promoter and the heterologous Psal and affected by the increase in copy number due to the presence of Ara. On the other hand, addition of Sal and Ara did not affect the high MIC values for Cem or Ctr obtained with the CemC19 derivatives. CEMC19 β-lactamase is oriented opposite to Psal and, consequently, its transcription is only controlled by its own promoter. Interestingly, MICs of carbapenems against CemC19 derivatives are increased by addition of Sal and Ara, likely by the amplification of the plasmid copy number induced by Ara.

**Table 4:** Minimum inhibitory concentration (MIC) of MPO554 strain carrying mutant fosmids.

Fosmid	MIC (μg/ml)							
	CEM		CTR		MER		IMI	
	+ Sal + Ara	-Sal-Ara	+ Sal + Ara	-Sal-Ara	+ Sal + Ara	-Sal-Ara	+ Sal + Ara	-Sal + Ara
Empty Vector	0.5	0. 5	0.06	0.06	< 0.125	< 0.125	< 0.125	< 0.125
TN2-Ctr	**64**	**32**	**128**	**16**	**8**	**4**	**4**	< 0.125
CEMC18-Cem	**64**	**16**	**32**	**2**	**0.5**	< 0.125	< 0.125	< 0.125
CEMC19-Cem	**> 128**	**> 128**	**128**	**128**	**8**	**4**	**0.25**	< 0.125
CEMC19-Ctr	**> 128**	**> 128**	**128**	**128**	**8**	**4**	**0.25**	< 0.125

Bold values correspond to fosmids and conditions showing resistance to antibiotics.

### Mutations increased β-lactamase activity without changes in the resistance genes

To identify mutations responsible of the phenotype, the four mutant fosmids were sequenced. Interestingly, none of them presented mutations in the β-lactamase coding sequence. CEMC19-Cem and CEMC19-Ctr had a single nucleotide insertion in a region that could correspond to the *E.coli* Shine-Dalgarno sequence. CEMC19-Cem had an Adenine and CEMC19-Ctr a Thymine, 7 and 10 bp upstream of the β-lactamase start codon, respectively. Surprisingly, the CEMC18-Cem fosmid had only a base substitution of a G to A transition 1184 bp upstream of β-lactamase translation initiation codon, that results in a silent mutation, TTG to TTA, at codon 233 of a gene homologous to *ftsI*, located immediately upstream of the β-lactamase ORF. Both genes are part of a three genes operon and expression of CEMC18 β-lactamase gene could be connected to that of the upstream gene by a translational coupling mechanism, since the start codon of the β-lactamase overlaps with the stop codon of the upstream gene. TN2-Ctr had an insertion of 1326 bp located 60 bp upstream of the β-lactamase ORF translation initiation codon. The inserted DNA corresponds to an IS4 family insertion sequence. This mutant also has a 12 bp duplication of the target DNA flanking the IS sequence. Members of this IS family carry outwards-directed promoters and have been associated with increased expression of different antibiotic resistance genes^33,34^. Resistance also raised in the presence of Ara and Sal, presumably by the increase in the copy number and consequently, in gene dosage, and by transcription from Psal subjected to antitermination.

**Figure 3:**
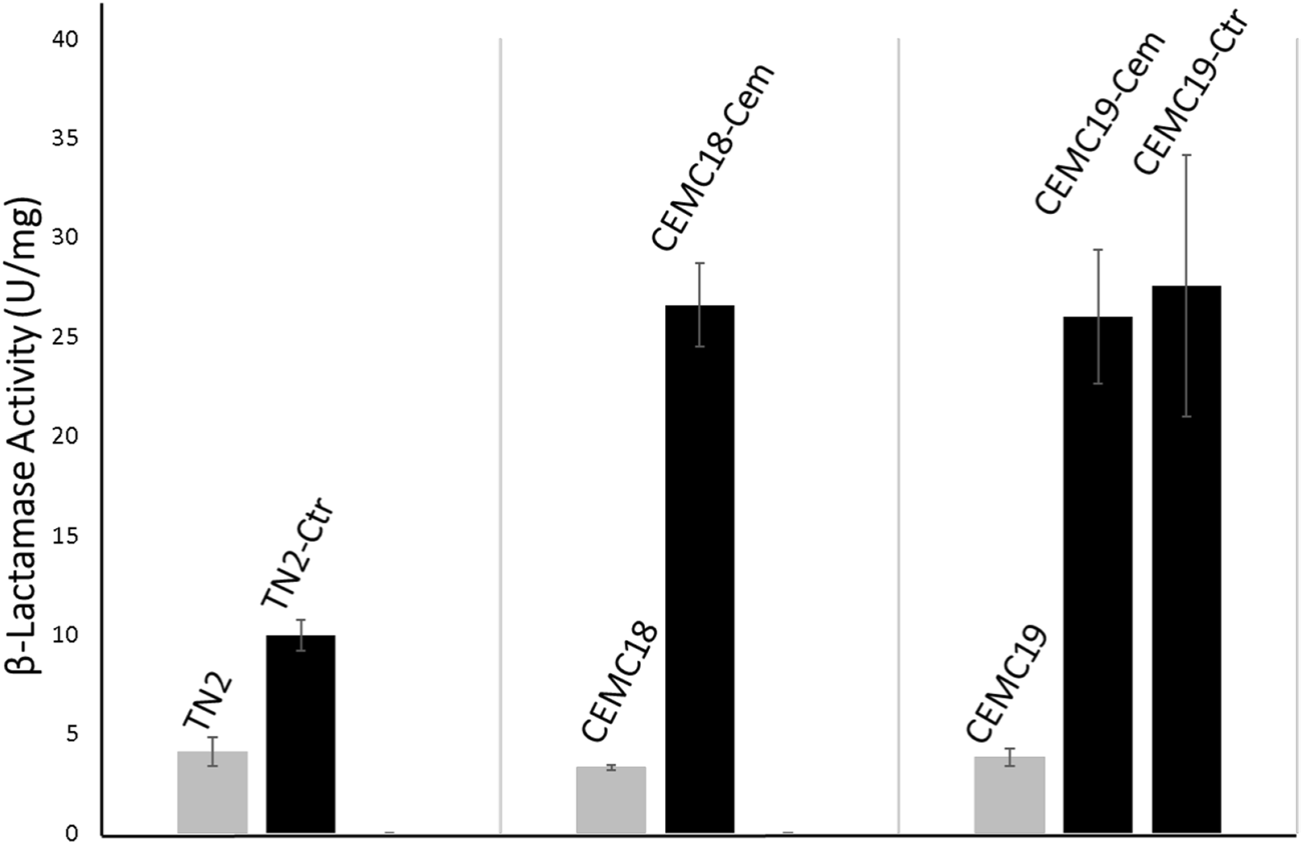
β-lactamase activity conferred by fosmids TN2, CEMC18, CEMC19 and their corresponding derivatives obtained by experimental evolution. Activity was measured as hydrolysis of nitrocefin. All data represent the average of three independent assays. Error bars indicate standard deviations.

Finally, to test whether mutations lead to changes in β-lactamase activities, we assayed them in crude extracts prepared from the strains harbouring these fosmids. As shown in Fig. 3, all mutant fosmids showed an increase in β-lactamase activity as compared to the wild type ones that ranged from 2.5 (TN2) to eightfold (CEMC18-Cem). Since mutations do not affect their coding regions, the higher resistance level displayed by mutant fosmids must be due to an increase in the β-lactamase expression.

## Discussion

AMR is a complex issue that threatens human and animal health and world economy. A recent estimation predicts that, if actions are not taken, 10 million people would die every year due to infections by AMR bacteria by 2050^35^. The severity of the matter has led the World Health Organization (WHO) to include AMR as one of the top ten threats to global health in 2019^36^, and different strategies underline the importance of combating the AMR from a One Health approach, holistic and multifactorial, to achieve optimal health for humans, animals and environment^37–39^. Provided that AMR emerging in humans, animals and environment can spread from one to the other, studying the occurrence of AMRs in environments other than clinical settings become a key strategy.

In this context, we have screened two metagenomic libraries constructed with bacterial DNA from oil contaminated soils to search for genes that confer resistance to antibiotics of restricted use in hospitals. Generally, oil contamination provokes a reduction of bacterial diversity and an increase of the abundance of degradative species^40–42^. Unlike other type of soils, oil contaminated soils are not expected to be enriched in antibiotic resistance genes, particularly, to those antibiotics used in this work. However, we have found genes coding for efflux pumps and, more interestingly, class D and subclass B1 and B3 β-lactamases coding genes that confer resistance to cephalosporins and carbapenems, some of them being last resort antibiotics used in hospitals. Interestingly, with the exception of TN2, which is related to CAU-1, the remaining β-lactamases branch in the same group of enzymes which have been found in clinical isolates^22,43,44^. This result highlights the convenience of extending this type of search to different types of soil or environments.

Conversely, we could not find any gene conferring resistance to non β-lactam antibiotics. Although it has been proposed that resistance genes related to aromatic antibiotics, such as fluoroquinolones, could be enriched in soils contaminated with polycyclic aromatic hydrocarbons^45,46^, we did not find genes providing resistance to Nor or Ofl. There is no clear explanation as to why only β-lactam resistance genes have been found. The absence of Nor, Ofl or Col resistance genes, could be due to the fact that soils used in this work, sand from a beach and soil from a refinery, could be particularly poor in biodiversity. In addition, β-lactamases are among the most widely used antibiotics for the treatment of infections and β-lactamases are one of the groups of resistance genes that have been co-selected with metal tolerance genes in previous works. However, further investigation is needed to clarify this issue. On the other hand, although numerous metagenomic analyses have been performed on different environmental samples, little is known about the presence and distribution of ARGs in oil-contaminated soils. Moreover, despite the co-selection of AMR determinants and metal tolerance genes described in some oil contaminated soils^26,27^, we did not identify, by BLAST analysis, metal tolerance genes except those encoding efflux pumps components. It should be noted that we have not detected sequences coding for plasmidic functions in any of the isolated fosmids, suggesting that metagenomic DNA likely correspond to chromosomic DNA. Given that the environmental DNA cloned in fosmids has an average size of about 30 kb, the probability of finding chromosomal fragments carrying genes conferring resistance to both metals and antibiotics is very low. This is probably the reason why we have also failed to find specific genes involved in the metabolism or detoxification of compounds associated with these polluted environments. Therefore, our results, based on a functional metagenomic approach, do not demonstrate a co-selection/cross-resistance between ARGs and metal tolerance genes.

Interestingly, BLAST analysis showed that all homologous resistance genes selected in previous works come from *Proteobacteria,* which is consistent with the fact that most of the human bacterial pathogens belong to this phylum^47^, and this taxonomic proximity could allow horizontal gene transfer of resistance genes to pathogenic bacteria. In this regard, the presence of mobile genetic elements in one of the fosmid that could contribute to the propagation of AMR determinants from the original microorganism is also noteworthy. In addition, IS4 family elements, such as the one found in CEMC18, could also be involved in increasing the level of resistance through expression of resistance genes from its outwards promoters, as demonstrated in our experimental evolution assays and elsewhere^33,34^.

Functional metagenomics studies have shown that transcription initiation from foreign promoters is an major limiting step, and different plasmid vectors and host strains have been developed to drive transcription of metagenomic genes (reviewed in^48^). In this work, we have used and efficient expression system based in a Sal inducible promoter, the lambda phage N antitermination system and the control of the fosmid copy number by the presence of Ara^29^. Although the *E. coli* host strain is able to efficiently transcribe AMR determinant promoters of some fosmids (CEMC6 and CEMC19), Sal and Ara are required in other fosmids to increase the resistance level or to display the resistance phenotype. These results emphasize the importance of using alternative and efficient expression systems to search for AMR determinants by metagenomic functional analysis. This system has allowed detection of these genes even when they confer resistance to low concentrations of antibiotics, including *bona fide* AMR determinants such as β-lactamases, which demonstrates the relevance of performing the screens using the minimum concentrations of antibiotics that prevent colony growth. The low resistance level of AMR genes could be due to a low concentration of antibiotics present in soil^31^ or inefficient transcription and/or translation in *E.coli*. This correlates with the “operational definition of antibiotic resistance gene” described by Martinez et al.^49^, in which a resistance gene allows a strain to grow in the presence of higher concentrations of a determined antibiotic than its parental strain, although these concentrations are much lower than epidemiological MICs. In this context, efflux pumps selected in this work are capable of providing resistance to low concentrations of antibiotics by themselves without the involvement of other AMR determinates, such as β-lactamases. Unlike the latter, efflux pumps are not expected to provide high resistance to antibiotics by increasing their expression because that could negatively affect the membrane structure and consequently bacterial fitness. However, the importance of these determinants must be considered since they could contribute to allow survival of pathogens growing in sublethal antibiotic concentrations until these bacteria can subsequently increase their resistance level by mutation or acquisition of additional resistance genes by horizontal gene transfer.

After experimental evolution assays, we obtained four fosmids with increased resistance level after only a few generations and due to single mutations, demonstrating how fast a bacterial strain can become resistant to these antibiotics once they bear the appropriate resistance gene. Different β-lactamases, such as CTX-M or TEM-1, have been subjected to experimental evolution to obtain new enzyme variants in the literature^50,51^, although, in our hands, none of the mutant fosmids with increased resistance level present mutations in their β-lactamase ORFs. Therefore, these β-lactamases sequences are already sufficiently effective to provide high resistance level when they are expressed at an appropriate level. The location of mutations present in CEMC19-Ctr and CEMC19-Cem, outside the coding region, indicates that the mutations could increase the amount of β-lactamase by improving its translation rate due to changes in their Shine Dalgarno sequences. The TN2-Ctr fosmid has an IS4 family insertion sequence upstream of the β-lactamase ORF, whose outwards-oriented promoter could lead to an increase in transcription rates and β-lactamase activity. By contrast, so far we do not have a clear explanation of the phenotype of CEMC18-Cem mutant fosmid. The only difference between mutant and wild type sequences is a silent mutation in the gene upstream of the β-lactamase gene, *ftsI*. Interestingly, *ftsI* codes for PBP3, a target of β-lactams. Mutations in this gene have been associated with β-lactam resistance in different species and genetic adaptation to growth under different conditions^52–54^. Since expression of *ftsI* and CEMC18 β-lactam genes could be connected by a translational coupling mechanism, an improvement in the translation efficiency of the upstream mutant gene could explain the increase in the expression of CEMC18-Cem β-lactamase. Since both, the mutant and wild type codons are equally used by *E. coli*, this improvement of expression could be due to other factors, for instance, by changes in RNA degradation rates^55^ or in bacterial fitness^56^ but further research is needed to determine the cause. Regardless the mechanism, what these results indicate is that a single nucleotide insertion or a single nucleotide substitution, which emerge in just one generation by a single mutation event is sufficient to increase the resistance level more than 200-fold, an increase that could make the bacteria insensitive to the therapeutic doses of the β-lactam antibiotic. On the other hand, the results presented here do not allow conclusions to be drawn about the expression levels of these AMR determinants and the corresponding levels of resistance they confer on the original strains in their own environments. However, experimental evolution assays show that, at least in the four cases cited above, the low levels of resistance conferred by the metagenomic DNA to the *E.coli* host are the result of insufficient transcription or translation of these genes.

Taken together, our results confirm the existence of relevant AMR determinants in types of soil not expected to be specially enriched in genes conferring resistance to antibiotics used in clinical settings and, additionally, they show the ease their genetic contexts can change to acquire higher resistance levels. The knowledge of the presence and evolution of genes conferring resistance to exclusive clinic antibiotics in different environments could help to solve a problem of general concern that requires urgent solutions.

## Methods

### Bacterial strains, growth conditions, plasmids and oligonucleotides

Bacterial strains used in this work and their genotypes are summarized in Table S1. Cells were grown in Luria–Bertani (LB) broth, incubated in tubes or flasks with shaking (180 r.p.m) at 37 °C, except for the screening and selection of fosmids conferring antibiotic resistance, which were carried out at 30 °C. When required, media was supplemented with the following antibiotics or compounds (Sigma-Aldrich): 12.5 μg mL^−1^ chloramphenicol (Cm), 15 μg mL^−1^ nalidixic acid (Nal), 25 μg mL^−1^ kanamycin (Km), 100 μg mL^−1^ ampicillin (Ap), 10 μg mL^−1^ gentamicin (Gm) and 20 μg mL^−1^ rifampicin (Rif). In the screens and experimental evolution assays, we used the following antibiotics: 0.75, 1 and 1.25 μg mL^−1^ imipenem (Imi), 0.1, 0.2 and 0.3 μg mL^−1^ meropenem (Mer), 1.5–55 μg mL^−1^ cefixime (Cem), 0.15–50 μg mL^−1^ ceftriaxone (Ctr), 0.08 μg mL^−1^ norfloxacin (Nor), 0.5 μg mL^−1^ ofloxacin (Ofl), 0.3 μg mL^−1^ levofloxacin (Lev) and 5 μg mL^−1^ colistin (Col). The copy number of the fosmids was increased by the addition of 1 mM arabinose (Ara) and 1 mM salicylate (Sal) was used as an inducer for increasing of transcription levels.

All DNA manipulations were performed according to standard protocols^57^. Antibiotic resistance genes were subcloned into pMPO579 together with about 500 pb of their respective 5’ upstream region. Efflux pump coding sequence of MERC5 was PCR amplified using primers FwEcoEPAB7 and RvEPAB7. The PCR product was digested with EcoRI and cloned into pMPO579 digested with EcoRI and PmlI to generate plasmid pMPO1706. Likewise, efflux pump ORFs of CTRC-R2 was PCR amplified with FwERV-R2 and Rv-R2 primers and cloned into PmlI site of pMPO579 to construct the plasmid pMPO1707. β-lactamase coding sequence of CEM4 was amplified by using FwEcoB3A1 and RvHindB3A1 primers, PCR product digested with EcoRI and HindIII and cloned into the same restriction sites of pMPO579 to construct pMPO1708. The same strategy was followed to clone CEMC18 and CEMC19 β-lactamase coding sequences into pMPO579 to generate pMPO1710 and 1711 respectively, but using the primers FwCEMC18Sph, RvCEMC18Hind, FwCEMC19Sph and RvCEMC19Hind and digestion with SphI and HindIII enzymes. These oligonucleotides, as well as those used to amplify Extended Spectrum β-Lactamases, are described in Table S2.

### Identification of clones conferring resistance to antibiotics

Two metagenomic libraries from oil contaminated soils previously constructed in our laboratory were screened to search for antibiotic resistance genes. One soil comes from a shore (Punta San García, Cádiz, Spain)^29^ and contains 54,000 independent clones (2 Gb of metagenomic DNA). The second soil comes from a parcel contiguous to a refinery plant (Huelva, Andalucía, Spain), with 185,000 different clones (6.5 Gb)^30^. Metagenomic DNA was extracted from 40 g of each soil and fragments, with an average size of 35 kb, and were cloned in the fosmid vector pMPO579. Metagenomic libraries, maintained in the strain EPI300-T1^R^ (Epicentre)^29^, were transferred by triparental matings^58^ to strain MPO554Nal^R^, which allows heterologous gene transcription of the metagenomic DNA, with DH5α/pRK2013 as the helper strain. Conjugative matings were performed on LB-agar without antibiotic selection and incubated overnight at 37 °C. Then, the mating mixtures were plated on LB-agar with Cm and Nal for transconjugants selection, one of the antibiotics used in clinical settings, Sal for inducing heterologous expression and Ara for increasing the copy number of the fosmid. Plates were incubated at 30 °C for 48 h. About 10^8^ transconjugants were screened with each antibiotic and, in those screens where colony growth were obtained, 20–50 clones were selected for further analysis.

### Antibiotic susceptibility assay

Minimum Inhibitory Concentration (MIC) assay was performed following the protocol already described^59^ according to the indications of the Clinical and Laboratory Standards Institute^60^. It was done in 96-well plate which contained Mueller–Hinton broth, the specified antibiotic under study at a concentration according to a twofold serial dilution from the first well, and ~ 10^5^ CFU of a strain carrying a metagenomic clone, in a final volume of 200 μL per well. In order to determinate if the resistance of these fosmids depends on an increase in the expression of resistance genes, tests were conducted in the presence or absence of Ara and Sal compounds. Furthermore, Cm was used to maintain the fosmid. The microplates were incubated at 30 °C for 2 days.

### Preparation of crude β-lactamase

20 mL of an exponential culture (OD_600_ = 0.6–0.8) was centrifuged, washed and resuspended in 100 μL of 100 mmol L^−1^ potassium phosphate buffer (pH 7). The cell suspension was sonicated in an ultrasonicator for 3 ranges of 5 s each in an ice bath. The generated suspension was centrifuged at 13,000 rpm for 2 min at 4 °C and the supernatant was used as an extract of β-lactamases.

### β-lactamase activity assay

The enzymatic activity of crude extracts of each β-lactamase was determined by the hydrolysis of the nitrocefin substrate^61^. The assay was performed in a 96 well plate and consisted in a 100 μL reaction mixture of 100 mmol L^−1^ potassium phosphate buffer, 40 nm nitrocefin and 25 μL extract β-lactamase, if it provided from the original fosmid, or 4 μL or 10 μL, if the extract provided from the improved fosmid. The reaction was measured by fluorescence at a wavelength of 390 nm for 2 min. Enzymatic activity of β-lactamase was defined as the amount of enzyme that was able to degrade 1 nmol of nitrocefin, defined in terms of units per mg of extract protein. Protein concentration was determined by Bradford method^62^.

### DNA sequencing and data analysis

Metagenomic CEMC6 clone was sequenced and assembled by MicrobesNG using Illumina technology and SPAdes v.2019 assembly method. Sequencing and assembly of the rest of fosmids were performed by STAB VIDA by using Illumina technology and DLC Genomics Workbench v.10.1.1 respectively. The structural annotation of fosmids were performed using PROKKA^63^.

Multiple-sequence alignment was performed with MEGAX software^64^, using MUSCLE as the alignment method. The Maximum Likelihood method was used to estimate the phylogenetic tree, following the best model indicated by MEGAX (WAG + G + I) and selecting the Partial Deletion option in the Gap/Missing Data Treatment parameter. Additionally, it was run a bootstrap analysis, choosing a No. of Bootstrap Replicated of 100^65^.

The online interface EMBOSS explorer (http://www.bioinformatics.nl/cgi-bin/emboss/) was used to calculate the fractional GC content of nucleic acid sequences, and to generate GC frequency plots.

To assess potential mechanisms of ARG mobility, genetic mobile elements, such as transposons and/or integrons were identified by using BLAST as the target database, and default settings.

### Experimental evolution

Isolated colonies of the strains UPO1, UPO2, CEM6, CEMC18 and CEMC19, as well as MPO554Nal^R^ /pMPO579 (the control), were inoculated in 3 mL of LB broth supplemented with Cm. The experimental evolution assays started inoculating 50 μL of the initial overnight cultures into 5 mL of LB supplemented with Cm, Ara and Sal, and with 0.15 μg mL^−1^ ceftriaxone or 1.5 μg mL^−1^ cefixime, respectively. Culture tubes were incubated at 30 °C and, every 2–4 days and during 2 months approximately, each culture was diluted 1:100 in the same medium, but gradually increasing the concentration of the respective β-lactam antibiotic. Subcultures were performed when culture growth reaches stationary phase, which was achieved at different times and at different concentrations depending on the fosmid and antibiotic. Likewise, the assay was stopped when a final antibiotic concentration was at least 20 times higher than the initial concentration.

### Nucleotide sequence accession numbers

The nucleotide sequences described in this work are available under GenBank accession numbers: MZ0638004 (CEMC6), MZ0638005 (CEMC18), MZ0638006 (MERC5), MZ0638007 (CTRC-R2), MZ079603 (CEMC19).

## Supplementary Information

Supplementary Information.

## Data Availability

All data generated or analysed in this study are included in this article.
