# Supplementary material for: Detection by metagenomic functional analysis and improvement by experimental evolution of β-lactams resistance genes present in oil contaminated soils
Source: Sci Rep. 2022 Jun 29;12:10059. doi: 10.1038/s41598-022-13883-x (PMC9243250; doi:10.1038/s41598-022-13883-x)
Supplement: Supplementary file 1 — Supplementary Information. [file 41598_2022_13883_MOESM1_ESM.docx]

**Detection by metagenomic functional analysis and improvement by experimental evolution of β-lactams resistance genes present in oil contaminated soils**

M. Teresa Álvarez, Laura Zarzuela, Eva M. Camacho, Eduardo Santero and Amando Flores*.

Centro Andaluz de Biología del Desarrollo/ CSIC/ Universidad Pablo de Olavide/ Junta de Andalucía. Departamento de Biología Molecular e Ingeniería Bioquímica, Seville, Spain

**Corresponding author: Amando Flores**

**Supplementary Table S1** Strains and plasmids

|  | Characteristics | Reference |
| --- | --- | --- |
| ***E. coli* strains** |  |  |
| *DH5α* | *F^–^ φ80lacZΔM15 Δ(lacZYA-argF)U169 recA1 endA1 hsdR17(rK^–^, mK^+^) gal– phoA supE44 λ^–^ thi-1 gyrA96 relA1* | (1) |
| EPI300-T1R | *F^–^ mcrA D(mrr-hsdRMS-mcrBC) (StrR) φ80dlacZDM15 ΔlacX74 recA1 endA1 araD139 Δ (ara, leu)7697 galU galK l2 rpsL nupG trfA tonA dhfr* | Epicentre |
| MPO554 Nal^R^ | *EPI300-T1R Δtrg:: nahR/Psal–gene N* Nal^R^ | (2) |
| MPO554 Gm^R^ | *EPI300-T1R Δtrg:: nahR/Psal–gene N* Gm^R^ | This work |
| **Plasmids** |  |  |
| pMPO579 | Cm^R^, fosmid expression vector | (2) |
| pRK2013 | Km^R^, helper in triparental matings | (3) |
| pMPO1706 | pMPO579 with efflux pump of MERC5 | This work |
| pMPO1707 | pMPO579 with efflux pump of CTRC-R2 | This work |
| pMPO1708 | pMPO579 with MBL of CEM4 | This work |
| pMPO1709 | pMPO579 with MBL of CEMC6 | This work |
| pMPO1710 | pMPO579 with β-lactamase of of CEMC18 | This work |
| pMPO1711 | pMPO579 with MBL of CEM19 | This work |

**Supplementary Table S2** Primers used in this work

| **Primers used for AMR genes subcloning** | | |
| --- | --- | --- |
| **Target fosmid** | **Primer sequence** | **Reference** |
| MERC5 | FwEcoEPAB7: ATGCGAATTCCGGCGAATCCACGCCGGAAATGC | This work |
|  | RvEPAB7: AGGCCGCGCCTTTACAGGCC |  |
| CTRC-R2 | FwERV-R2: GATATCGCCGGTTTGTTGGCC | This work |
|  | Rv-R2: GCGGCCGCATGGCGAATAC |  |
| CEM4 | FwEcoB3A1: TGCAGAATTCACTGGGCGATCCAACCGTCC | This work |
|  | RvHindB3A1: TGCAAAGCTTCTATTTCGCCGCCCGTTCTTTC |  |
| CEMC6 | FvCEMC6Sph: TGCAGCATGCAAACGGGAATCGTCAAAG | This work |
|  | RvCEMC6Hind: TCGAAAGCTTAAAGTATCACAACTTTTAG |  |
| CEMC18 | FwCEMC18Sph: TACGGCATGCATTAAGGTGTCGCAAGAGAC | This work |
|  | RvCEMC18Hind: TAGCAAGCTTATGCGGTCGTAGTGATG |  |
| CEMC19 | FwCEMC19Sph: CGATGCATGCTCTCCAGAAGTACCAGTAC | This work |
|  | RvCEMC19Rv: TGACACGCGTACCTGGAACATCGGAGAAG |  |
|  |  |  |
| **Primers used for detection of β-lactamases** | | |
| **Target gene** | **Primer sequence** | **Reference** |
| TEM | TEM front P1: GCGGAACCCCTATTT G | (4, 5) |
|  | TEM-C-R-ny: ACCAATGCTTAATCAGTGAG |  |
| SHV | SHV OS5: TTATCTCCCTGTTAGCCACC | (6) |
|  | SHV OS6: GATTTGCTGATTTCGCTCGG |  |
| CTX | CTX M U1: ATGTGCAGYACCAGTAARGTKATGGC | (7, 8) |
|  | CTX-M-U-2new: TGGGTRAARTARGTSACCAGAAYSAGCGG |  |
| blaKPC | KPC-Fm: CGTCTAGTTCTGCTGTCTTG | (9) |
|  | KPC-Rm: CTTGTCATCCTTGTTAGGCG |  |
| OXA 1, 4, 30 | MultiTSO-O_for: GGCACCAGATTCAACTTTCAAG | (10) |
|  | MultiTSO-O_rev: GACCCCAAGTTTCCTGTAAGTG |  |
| OXA 48 | MultiOXA-48_for: GCTTGATCGCCCTCGATT | (10) |
|  | MultiOXA-48_rev: GATTTGCTCCGTGGCCGAAA |  |
| OXA 23 | OXA-23f GATCGGATTGGAGAACCAGA | (11) |
|  | OXA-23r ATTTCTGACCGCATTTCC AT |  |

1. Hanahan D. 1983. Studies on transformation of Escherichia coli with plasmids. J Mol Biol 166:557–580.

2. Terrón-González L, Medina C, Limón-Mortés MC, Santero E. 2013. Heterologous viral expression systems in fosmid vectors increase the functional analysis potential of metagenomic libraries. Sci Rep 3.

3. Figurski DH, Helinski DR. 1979. Replication of an origin-containing derivative of plasmid RK2 dependent on a plasmid function provided in trans (plasmid replication/replication origin/trans-complementation/broad host range/gene cloning). Proc Nati Acad Sc 76:1648–1652.

4. Olesen I, Hasman H, Aarestrup FM. 2004. Prevalence of β-lactamases among ampicillin-resistant Escherichia coli and Salmonella isolated from food animals in Denmark. Microb Drug Resist 10.

5. Moodley A, Guardabassi L. 2009. Transmission of IncN plasmids carrying blaCTX-M-1 between commensal escherichia coli in pigs and farm workers. Antimicrob Agents Chemother 53:1709–1711.

6. G A, M R, A P. 1997. Substitution of alanine for aspartate at position 179 in the SHV-6 extended-spectrum beta-lactamase. FEMS Microbiol Lett 152:163–167.

7. H H, D M, K V, I O, FM A. 2005. beta-Lactamases among extended-spectrum beta-lactamase (ESBL)-resistant Salmonella from poultry, poultry products and human patients in The Netherlands. J Antimicrob Chemother 56:115–121.

8. RS H, M M, C K, RL R, SV D, C K, H H, M C, D M, J T, FJ A, FM A. 2009. Emergence of multidrug-resistant salmonella concord infections in Europe and the United States in children adopted from Ethiopia, 2003-2007. Pediatr Infect Dis J 28:814–818.

9. L P, TR W, V C, P N. 2011. Multiplex PCR for detection of acquired carbapenemase genes. Diagn Microbiol Infect Dis 70:119–123.

10. C D, A DC, D D, C F, G A. 2010. Development of a set of multiplex PCR assays for the detection of genes encoding important beta-lactamases in Enterobacteriaceae. J Antimicrob Chemother 65:490–495.

11. Hofko M, Mischnik A, Kaase M, Zimmermann S, Dalpke AH. 2014. Detection of Carbapenemases by Real-Time PCR and Melt Curve Analysis on the BD Max System. J Clin Microbiol 52:1701.

**Genebank accession numbers of the previously sequenced β-lactamases included in the phylogenetic tree**

IMP-1, ADI87504; SIM-1, AAX76774; KHM-1, BAH16555; DIM-1, L7X1X1; TMB-1, E7BCS5; GIM-1, CAF05908; BcII, PO4190; BlaB1, AF189298; NDM-1, CAZ39946; VIM-1, CAC35170; CcrA, Q2KK85; CAU-1, AJ308331; GOB-1, AAF04458; FEZ-1, CAB96921; L1, ABO60992; POM-1, EU315252; AIM-1, AM998375; THIN-B, CAC33832; SMB-1, AB636283; OXA23, AJ132105; OXA40, AF509241; OXA10, U37105; OXA1, J02967; LCR1, X56809; OXA2, X07260; OXA20, AF02460.

**Supplementary Figure S1**

Amino acid alignment of isolated subclass B1 and B3 MBLs with previously known MBLs. Zinc-binding amino acids are indicated by a red star on top. [*]: positions with a fully conserved residue. [:]: conservation between amino acids of very similar properties. [.]: conservation between amino acids of weakly similar properties.


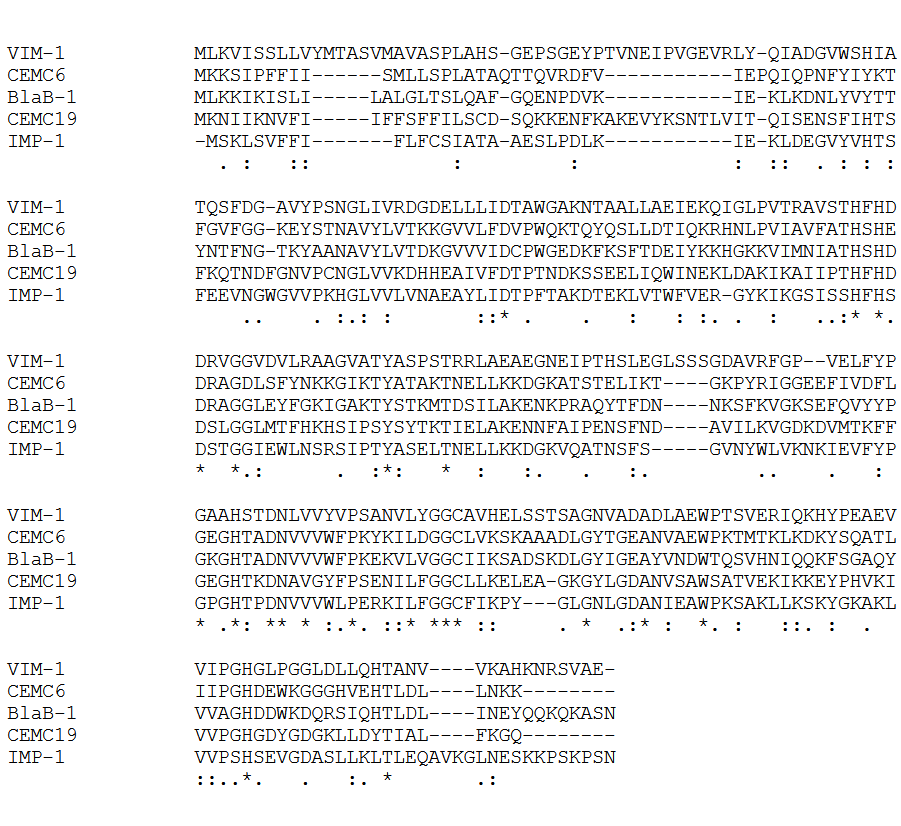


**116**

**263**

**196**

**221**


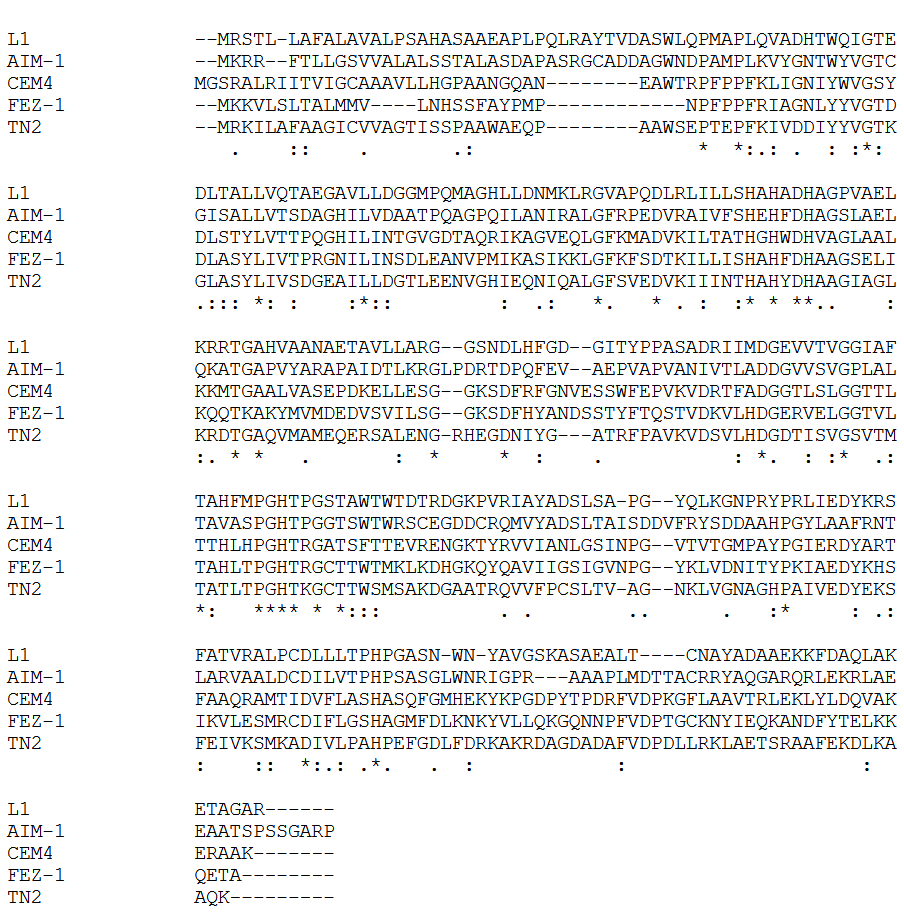


**116**

**196**

**263**
